# A Review of the Effects of Olive Oil-Cooking on Phenolic Compounds

**DOI:** 10.3390/molecules27030661

**Published:** 2022-01-20

**Authors:** Roberto Ambra, Sabrina Lucchetti, Gianni Pastore

**Affiliations:** CREA—Research Center for Food and Nutrition, Via Ardeatina 546, 00178 Rome, Italy; sabrina.lucchetti@crea.gov.it (S.L.); giovanni.pastore@crea.gov.it (G.P.)

**Keywords:** EVOO, vegetables, phenolic compounds, thermal treatment, processing techniques, bioaccessibility, bioavailability

## Abstract

The fate of phenolic compounds in oil and food during cooking vary according to the type of cooking. From a nutritional point of view, reviews largely suggest a preference for using extra-virgin olive oil at a low temperature for a short time, except for frying and microwaving, for which there appears to be no significant advantages compared to olive oil. However, due to the poorly pertinent use of terminology, the different protocols adopted in studies aimed at the same objective, the different type and quality of oils used in experiments, and the different quality and quantity of PC present in the used oils and in the studied vegetables, the evidence available is mainly contradictory. This review tries to reanalyse the main experimental reports on the fate, accessibility and bioavailability of phenolic compounds in cooking oils and cooked vegetables, by considering different cooking techniques and types of oil and foods, and distinguishing experimental findings obtained using oil alone from those in combination with vegetables. The re-analysis indicates that incomplete and contradictory observations have been published in the last few years and suggests that further research is necessary to clarify the impact of cooking techniques on the phenolic compounds in oil and vegetables during cooking, especially when considering their nutritional properties.

## 1. Introduction

Different culinary practices are involved in heating food with vegetable oils. Among the different techniques mentioned below (Section 4), the most widespread is deep-frying, where food is entirely submerged in hot oil, inducing a process that rapidly modifies the nutritional and organoleptic properties of oil and food, normally improving those of the latter and worsening those of the former. This cooking technique has been widely used since Ancient Egypt, and oral tradition suggests that the practice is as old as the invention of pots, more than six thousand years ago [1]. Its success, historically linked to the usage of oil obtained from Olea europaea var. sativa L. fruits in other practices (lighting, religion and aesthetics), lies in the fact that it produces tastier and more durable properties in the resulting food, compared to raw food or other cooking procedures. Nowadays, even if some limitations have been introduced, especially by Mediterranean countries due to a possible link between fat consumption and obesity, oil-cooking is one of the most widely used food preparation processes worldwide [2].

Cooking with oil triggers a series of physico-chemical reactions, favoured by high temperatures, oxygen, water, and metals, which have lipids as their main targets, but also other fat components i.e., tocopherols, pigments, sterols, vitamins and phenols. Cooking with oil can yield molecules with cytotoxic, mutagenic and carcinogenic activities, affecting the cardiovascular system or generating systematic inflammation [3,4]. Interestingly, such effects are influenced by the interaction between ingredient molecules, antioxidants, and other bioactive molecules. Among those, and besides monounsaturated fatty acids, tocopherols and tocotrienols are mainly present in seed oils, while phenolic compounds (PC) may be particularly abundant in unrefined olive oils (simple phenols, secoiridoids, lignans, flavonoids, phenolic acids and alcohols), able to confer specific organoleptic features to foods and induce different reactivity to cooking oils. The ability of PC to prevent oxidative deterioration occurring in olive oil (OO) during heating was hypothesized more than 20 years ago [5]. More recently, PC in OO were shown to reduce the formation of acrylamide during potato frying [6]. 

Contradictory evidence is available on the fate and role of PC in oil and food during oil cooking procedures. A recent meta-analysis indicated that frying was the only cooking technique, along with steaming, that was not associated with statistically significant losses of PC from vegetables [7]. Some studies indicate losses from both food and oil, whereas others suggest some migration between oil and food. A partial explanation is that experimental cooking conditions, mimicking home ones, are hardly repeatable in and comparable between laboratories. Moreover, most works have studied the effects of heating at very long durations (repeated for even days), which are poorly representative of domestic conditions (short and unrepeated heating). 

Additionally, insufficient data are available on the physical aspects of food heating. Research on other vegetable molecules indicates that heating could induce a net increase in PC as a result of several mechanisms i.e., partial evaporation of moisture that increases concentration in the food matrix, migration toward media with different polarity, changes in the microstructure of vegetable tissues, breakage or softening of the rigid cell walls and other components of the vegetable cells, and the decomposition of molecules linked to fibre. A recent review [8] reported that total frying time is normally limited to the first phase of the water cycle, evaporation, with the inner temperature of food not exceeding 100 °C. However, to our knowledge, this assumption, made 20 years before [9], was never corroborated. Other experiments with different kinds of food products indicate that the main effects on PC content rely on food cooked in oil rather than on oil itself. The situation is complicated by the fact that different oil cooking techniques exist, each with a different influencing factor that, if not considered, can result in misleading conclusions regarding health effects, as recently recapitulated by Garcimartín and collaborators in a book chapter [10]. 

This review tries to reanalyse the reports on the fate and on the bioavailability of PC in cooking oils and cooked vegetables, considering the different culinary approaches and the types of oil and foods and focusing on experimental findings obtained by both using oil alone or in combination with vegetables.

## 2. Phenolic Compounds Classification

PC, very widespread in the plant world, are bioactive molecules essential in numerous functional processes [11]. Their common structural characteristics include at least a hydroxyl group linked to an aromatic ring, but PC can also be polymerized compounds with functional groups such as mono or polysaccharides, esters and methyl esters with one or more phenolic group [12].

Historically, PC have been classified taking into account various parameters. For example, in 1962 Swain and Bate-Smith classified PC considering their spread [13], in 2017 Basli considered uniquely their carbon skeleton [14], and in 2019 Tsimogiannis and Oreopoulou classified phenols in a much more specific way, subdividing them into six classes [15]: (1) molecules with a single benzene ring (C6 class); (2) molecules with a C6 ring and a linked chain composed of one to four or seven carbon atoms (C6-Cn class); (3) and molecules belonging to the previous class with the addition of one more benzene ring linked to the carbon chain (C6-Cn-C6 class). In respect to these groups, complex phenols are organized as follows: (4) dimers [(C6-C1), (C6-C3)_2_, (C6-C2-C6)_2_, (C6-C3-C6)_2_], (5) oligomeric compounds due to the condensation of dimers, (6) polymers, normally with the following structure [(C6)_n_, (C6-C3)_n_, (C6-C3-C6)_n_] (7) hybrid phenolics constituted of phenolics connected to other types of molecules, such as terpenes or lipids [15]. Below are presented only categories containing molecules that are discussed in the following paragraphs.

**Figure 1:**
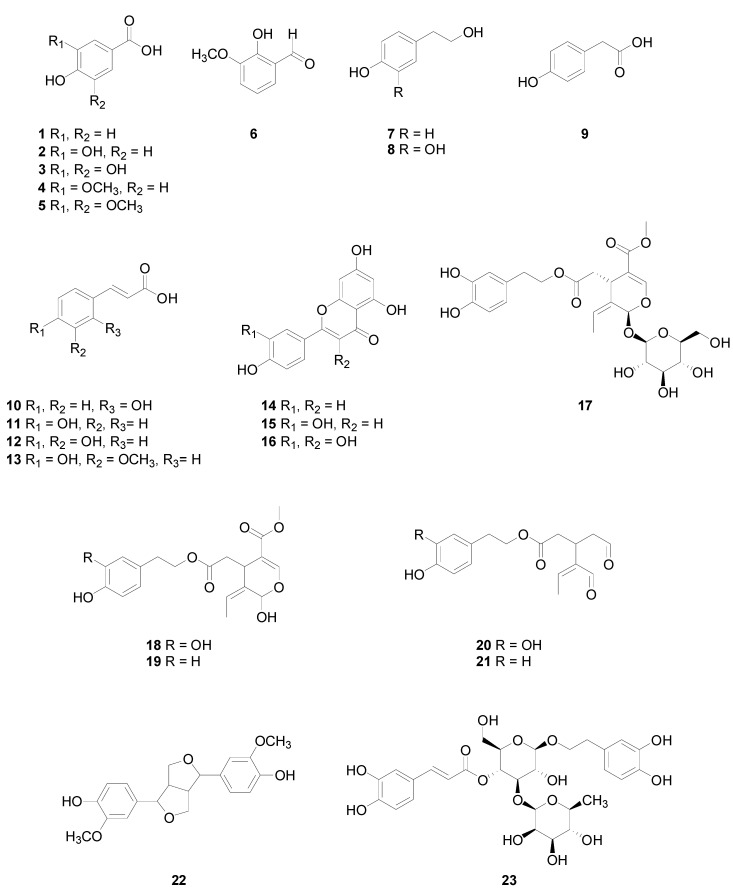
Phenolic compounds mentioned in figures. C6-C1 group: **1** *p*-hydroxybenzoic acid; **2** 3,4-dihydroxybenzoic acid; **3** gallic acid, **4** vanillic acids; **5** syringic acid; **6** *o*-vanillin. C6-C2 group: **7** tyrosol; **8** hydroxytyrosol; **9** *p*-hydroxyphenyl acetic acid. C6-C3 group: **10** *o*-coumaric acid; **11** *p*-coumaric acid; **12** caffeic acid; **13** ferulic acid. C6-C3-C6 group: **14** apigenin; **15** luteolin; **16** quercetin. Secoiridoids: **17** oleuropein, **18** 3,4-DHPEA-EA; **19** pHPEA-EA; **20** 3,4-DHPEA-EDA; **21** *p*-HPEA-EDA. Lignans: **22** pinoresinol; **23** verbascoside.

Gallic, *p-*hydroxybenzoic, 3,4-dihydroxybenzoic, vanillic, salicylic, ellagic and syringic, caffeic and ferulic acids are some examples of the C6-C1 group typically found in fruits, cereals, vegetables and teas, and interestingly, many of them are also characteristic of OO (Figure 1). The C6-C2 group (phenylethanoids) is scarcely present in plants but contains two compounds very characteristic of OO, namely tyrosol (Ty) and hydroxytyrosol (Hy) [16] (Figure 1). Largely distributed in the vegetal world are hydroxycinnamic acids, the main molecules of C6-C3 group (phenylpropanoids), and the more diffused are coumaric, caffeic and ferulic acids (Figure 1), often esterified with other molecules. Coumarins, also belonging to the C6-C3 group, can be found in a free status or coupled with sugars, such as heterosides and glycosides in many plant families. Among the different molecules included in the C6-Cn-C6 group worth mentioning are flavonoids (C6-C3-C6) (Figure 1), the most widespread PC in the vegetal world and largely present in the human diet. Flavonoids are mainly constituted by two aromatic rings linked through a 3−carbon bridge that often forms a heterocyclic ring. Of note are quercetin and kaemferols, widespread and very concentrated in capers and in red onion, the former is also present in OO, while the latter in cabbage, beans, tea, spinach and broccoli. Apigenin and luteolin, present in OO, are within flavones, while naringenin, abundant in the genus citrus, is among flavanones [17]. Within dimers of the C6-C3 group are lignans (Figure 1). Pinoresinol is a lignan found in sesame seeds and in OO [18]. Finally, worth mentioning are secoiridoids, olive oil-specific PC [19] originating from oleuropein and ligstroside, i.e., the oleuropein aglicone mono-aldehyde (3,4-DHPEA-EA), the ligstroside aglicone mono-aldehyde (*p-*HPEA-EA), the dialdehydic form of elenolic acid linked to Hy (3,4-DHPEA-EDA, or oleacin) and the the dialdehydic form of elenolic acid linked to Ty (*p*-HPEA-EDA, or oleocanthal) (Figure 1). 

## 3. Origin and Functions of Phenolic Compounds

Despite the enormous differences found among the group, all PC originate from only two distinct pathways, namely the shikimimate/phenylpropanoids and the acetate/malonate (polyketide) pathways [20]. Different approaches, such as knockout mutations and small RNA silencing in plant models, have determined the identification of regulatory genes, biosynthetic enzymes and metabolites [21]. PC constitute an important evolutionary and diversification strategy for plant terrestrial colonization, ensuring safety from both abiotic (hydric, thermal, ultraviolet radiation, heavy metals) and biotic (predators and pathogens) seasonal stresses [22], and functional roles in growth and reproduction (for example acting as attractors for pollinating insects through colours and sensory characteristics) [23]. 

Many in vitro and in vivo studies have demonstrated a positive relation between PC and human health as anti-inflammatory, antibacterial and, above all, as antioxidant compounds [24]. In particular, the antioxidant capacity of PC was related to their structural characteristics, through which they can donate hydrogen or electrons, or also chelate iron and copper metals, therefore suppressing radical formation due to the presence of metals. Each group of molecules express a different antioxidant capacity related to the number and position of the hydroxyl groups linked to the carboxyl functional group. Due to their widespread presence in fruits, vegetables and beverages, PC have received significant attention in the last few decades as sources of healthy molecules in a correct and healthy diet [25]. Worth mentioning are some functions of PC present in oils produced from olives, the main subject of this review, in particular the four secoiridoids that yield Ty and Hy and that originate from oleuropein and ligstroside, the oleuropein aglicone mono-aldehyde (3,4-DHPEA-EA), the ligstroside aglicone mono-aldehyde (*p-*HPEA-EA), the dialdehydic form of elenolic acid linked to Hy (3,4-DHPEA-EDA, or oleacin) and the the dialdehydic form of elenolic acid linked to Ty (*p-*HPEA-EDA, or oleocanthal) (Figure 1) [26].

With respect to Ty, Hy, syringic acid and verbascoside, the effects of which on human health have been recently reviewed [27], animal and cell models indicate the involvement of caspase 3 in antioxidant, antigenotoxic and proapoptotic activities [28,29]. Secoiridoids anticancer effects involve the modulation of redox, inflammatory and cell-regulatory factors (reviewed in [30]). In particular, pHPEA-EDA was shown to be able to selectively block cyclooxygenases [31], demonstrating that, together with inflammatory transcription factors, kinases and cytokines are also present in antimutagenic and anticancer downstream pathways of gallic, caffeic and coumaric acids, verbascoside and pinoresinol [32,33,34,35,36]. Using cell models, the anticancer activities of apigenin and luteolin have been linked to the microRNA-dependent downregulation of E2F1/3 transcription factors [37], and the use of rats confirmed luteolin’s properties in limiting the adverse effects of antitumorals [38,39]. Free radical scavenging or pro-apoptotic properties and ferulic, vanillic, syringic and 3,4-dihydroxybenzoic acids were demonstrated by in vitro studies [29,40], while animal models showed that restoration properties (e.g., on gut microbiota or asthma) by these molecules involved the modulation of several inflammation parameters and pro-inflammatory cytokines [41,42,43].

## 4. Are the Phenolic Compounds Influenced by Cooking?

During cooking, PC present in oils and foods are subjected to different chemical reactions that obviously depend on the cooking method technique used: deep-fat frying, sautéing (shallow/pan frying of vegetables in little amount of oil), roasting (air-baking of superficially oiled vegetables), air-frying with a small amount or no oil at all and microwaving and boiling with oil (cooking with water/oil mixes). Oil cooking reduces vegetable content in phytosterols, and reduction was linked to several variables, from length and type of technique used to oil unsaturation. Final products in deep-fat frying and roasting consist mainly in superficially browned food. In shallow frying, products range from flavoured tomato sauces for first dishes to eggplant Parmigiana, in which raw vegetables are greatly improved from a flavour point of view. As described below, on one hand, the presence of vegetables affects the content of PC in EVOO, on the other, cooking with EVOO may improve the uptake of PC, thanks to the transfer of molecules from oil to raw vegetables. Different cooking procedures are also related to the level of production of fried items: home, restaurant and industry. While domestic choices mainly favour tradition and health vs. efficiency and shelf life, industries are mainly oriented towards efficiency and price, while restaurateurs’ choices are more variable due to the huge diversity in the quality of food offered and the type of consumers targeted. For example, detrimental repeated frying is avoided in shallow frying as oil is used just once, with consequences on oil uptake. On the other hand, while restaurants tend to reuse and/or partially replenish deep-fat frying after oil cooling due to economic reasons, with effects on oil quality, industries mainly perform deep-fat frying in a continuous, but controlled, manner, in order to implement frying for consecutive hours or even days [44]. Finally, home deep-fat frying tends both to discard oil every time but also to reuse it, based on arbitrary criterion [2]. Very little is known on the effects on PC in industrial-level cooking, as practically all studies have been performed in laboratory simulations. 

### 4.1. Non-Olive Oils

Very few experiments have analysed the fate of PC in oils different from olive ones during heating. Nogueira-de-Almeida and Castro applied the same domestic conditions used for OO (200 °C for 6 min, in absence of food) to soybean and sunflower seed oils and evaluated changes in total PC (TPC) concentration [45]. TPC losses were higher for EVOO (20% loss) than for refined OO (8% loss) and complete (undetectable TPC) in both soybean and sunflower seed oils [45]. 

In the presence of food, the loss of TPC was complete following the deep frying (3 h at 170 °C) of fresh potatoes with a refined vegetable oil blend, consisting mostly of sunflower oil [46]. However, differently from the above publication, the loss of TPC was lower (by 42%) for EVOO than for a blend of refined and VOOs (53% loss) [46]. Some authors used the technique of spiking vegetable oils with defined amounts of PC (see also Section 4.3.1). This approach was used in a set of experiments published over 7 years by the Greek research group headed by Andrikopoulos, aimed at studying the impact of PC on the preparation of fried potatoes. Specifically, the authors enriched different oils (olive, sunflower, palm, soybean) with increasing the amounts of olive leaf extract [47,48,49,50]. Authors made an observation that still questions the validity of studies comparing the effects of cooking on oil PC. In fact, they observed that the efficiency of recovery of TPC from freshly spiked oils-before cooking-was very low and variable (from 38 to 72%), and also depended on the type of oil, i.e., higher for sunflower oil and lower, and not proportional with the amount spiked, for palm oil [47]. Additionally, the recovery of individual PC present in the leaf extract was affected. As a consequence, the recovery of TPC from oils, following the pan-frying of potatoes, barely correlated with the amount of spiked PC. Authors hypothesized a role of different chemical and physical properties of oils, but a clear corroboration is still lacking. Nonetheless, potatoes fried in phenol-spiked sunflower oil (but not palm oil) were more enriched in TPC (and individual PC, especially oleuropein) than those fried in phenol-spiked OO using the same frying conditions (201 gr of sliced potatoes fried at 175 °C for 6 min) [47], suggesting that sunflower oil could be more indicated than OO if one wants to enrich food with PC. However, the migration of PC was due to the food’s absorption of the cooking oil, which calls for nutritional considerations. Later, the authors reported that supplementation of oils with TPC also increased tocopherols transfer to potatoes by frying, which was again higher for sunflower vs. OO [48]. Unfortunately, even if the authors expressly declared that oils were chosen based on their different saturation levels (reporting the chemical analysis), they did not correlate nor discuss this with respect to this characteristic. Moreover, looking at their following publication analysing longer and repeated frying sessions effects [49], it appears that the higher enrichment achievable with sunflower, compared to OO, was temporary. Unfortunately, in this last publication, the authors did not test the effects on TPC. 

Santos and collaborators recently compared the enrichment in PC of potatoes fried by air-frying with different vegetable oils (canola, soybean, sunflower and olive) [51]. In contrast to the above set of experiments, sunflower oil was the only oil that did not increase the amount of PC in air-fried potatoes. In particular, even if potatoes cooked in OO had higher absolute content in PC (thanks to higher PC content of OO), the PC increase in air-fried potatoes was higher for canola, followed by soybean and olive oils, possibly because of different fatty acid content, suggesting again the opportunity to enrich with phenolic extracts non-OO oils rather than OO ones. However, even if the authors performed air-frying for longer cooking times (15–25 vs. 6 min for deep-frying), air-fried potatoes did not retain more fat and neither fats were more oxidized (as indicated by lower content of trans fatty acid in potatoes [51]), which is quite unbelievable, especially for those PC whose concentration was reported reduced by other authors (see Section 4.3.4). 

### 4.2. Effects of Oil Heating without Food

**Figure 2:**
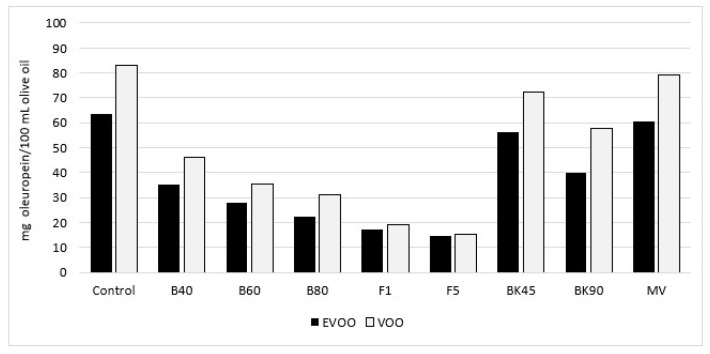
Impact of different heating processes on TPC of EVOO and OO in absence of foodstuff. Folin–Ciocalteu absolute quantification of TPC (mg oleuropein/100 mL of oil) in EVOO (black) and OO (white) subjected to boiling at 100 °C for 40, 60 and 80 min (B40, B60 and B80), heating at 180 °C for 1 or 5 h (F1 and F5), baking at 180 °C for 45 and 90 min (BK45 and BK90) and microwaving for 5 min at 500 W (MW). Picture from Goulas et al. [52].

Unfortunately, only a few studies have analysed the effects of heating in the absence of foods that can provide their own PC to EVOO or absorb PC of EVOO. Moreover, as mentioned above, cooking with oil at home involves heating using different processes that affect the fate of PC, possibly to different extents. The work by Goulas and co-workers is one of the few that recently tried to recapitulate the effects occurring in different heating processes: boiling (60 mL of oil in 400 mL of water for 40, 60 and 80 min), frying (actually heating, as 50 mL oil samples were poured into glass beakers and heated on heating plates at 180 °C for 1 or 5 h), hot-air oven baking (50 mL at 180 °C for 45 or 90 min) and microwaving (50 mL for 5 min at 500 W) (final temperature was not reported) [52]. The authors concluded that the only heating procedure that did not induce loss of TPC (estimated both by Folin–Ciocateau and by fluorometric assay) was microwaving, while the loss for baking was 11%, and boiling and frying induced relevant losses, up to near 75% for heating, time dependent for boiling but not for heating [52]. However, unfortunately, as can be noticed already from Figure 2 (reproduced from authors publication), the duration of the heat treatments was very different, even for similar procedures (boiling, frying and baking, please see the footnote), making it very difficult to compare the effects of different cooking methods. 

Most studies that have analysed the effects of heating on PC in EVOO in absence of foods used HPLC or MS. One significant exception is the work by Campanella and co-workers that, using a biosensor to monitor changes in oxygen concentration during the tyrosinase enzyme-catalysed oxidation of PC to quinone, studied the kinetics of the decay of TPC in EVOO (25 mL), artificially oxidized by exposure to heat (98, 120, 140, 160, or 180 °C) and constant air flow exposure for 3 min to 60 h [53]. The authors noticed the complete disappearance of TPC at 98 °C and 180 °C in, respectively, 60 h and 6 h, which is a notable difference. Moreover, they estimated the half-value concentrations (respectively, 55, 45, 25, 17 and 6 min) and concluded that the kinetic constant of the process at 180 °C is about 20 times higher than at 98 °C [53]. Importantly, the same group well correlated with the kinetics of the decay of TPC to that of the oil total antioxidant capacity, especially at 180 °C [54]. Unfortunately, the authors did not follow the decay of PC individually. More recently, Kishimoto et al. used a commercial tester (OxiTester) to compare TPC loss during heating and microwaving [55]. Although this method lacks the accurate validation of TPC quantification [56], the paper is discussed as it reported the accurate detection of temperature kinetics in both heating processes (see above, Section 4.2.1). 

A work used MRM tandem mass spectrometry assisted by isotope dilution to test the kinetics of the thermal decomposition of individual PC, i.e., labelled Tyrosol (Ty), Hydroxytyrosol (Hy), *p-*HPEA-EDA and 3,4-DHPEA-EDA, in OO samples treated at 90, 170 or 220 °C for 30, 90 or 150 min [57]. With respect to Hy and Ty, the authors reported the complete disappearance of the former and higher resistance for the latter, as more than 50% of the initial amount lasted till the end of the treatment after 150 min at 220 °C, possibly due to the absence of the catechol moiety [57]. With respect to secoiridoids, while losses after 30 min at 90 °C were neglectable, heating at 220 °C for 150 min caused “complete decomposition” without particular differences between Ty and Hy derivatives. However, it cannot be excluded that differences were not appreciated by the authors because the samples probably had very different compositions, as demonstrated by the huge differences reported for intermediate times and temperature treatments [57].

Accordingly, later, Carrasco-Pancorbo used separative techniques (HPCL and CE) to characterize the deterioration of EVOO at 180 °C (100 mL for 30, 60, 90, 120, 150 and 180 min), demonstrating faster degradation for Hy and its derivatives (3,4-DHPEA-EDA and 3,4-DHPEA-EA) compared to Hy-Ac, *p-*HPEA-EA and lignans [58]. Similar results were obtained by Daskalaki et al. who confirmed that incubation at 180 °C for 60 min induced about 38% reduction of the Ty derivatives and total loss of the Hy ones and reported that lignans are also stable at such high temperatures. Moreover, they demonstrated that heating at a lower temperature of 100 °C only minimally affected Ty and Hy derivatives (20% loss) [59]. 

Going back in time to one of the first publications that used HPLC to study the fate of PC in EVOO during heating in the absence of foodstuff, the work from Brenes et al. [60] deserves special attention. Authors checked PC in two monocultivar EVOOs (Picual and Arbequina) heated at 180 °C for 1.5 to 25 h. Although this publication is highly cited, it has some limitations that to our knowledge have never been noticed and thus are worth mentioning. Firstly, as data for shorter incubations of oil was reported incompletely, one cannot exclude that the “strange behaviour” of one of the two oils (authors reported a surprising early increase in *p-*HPEA-EDA concentration during the first 5 h of heating) could happen also in the other one. Secondly, authors concluded that the “disappearance rate depended on each individual compound and the olive cultivar” and correlated lower retention of PC to oil chemical stability, i.e., lower content in polyunsaturated fatty acids [60]. However, a deeper observation of the authors’ figures on Ty and Hy and their derivatives reveals that the two EVOOs analysed probably had very different amounts of TPC (that were actually not quantified). Such a difference regarding the content in polyunsaturated fatty acids (hypothesized by authors) could actually account for different losses in PC.

**Figure 3:**
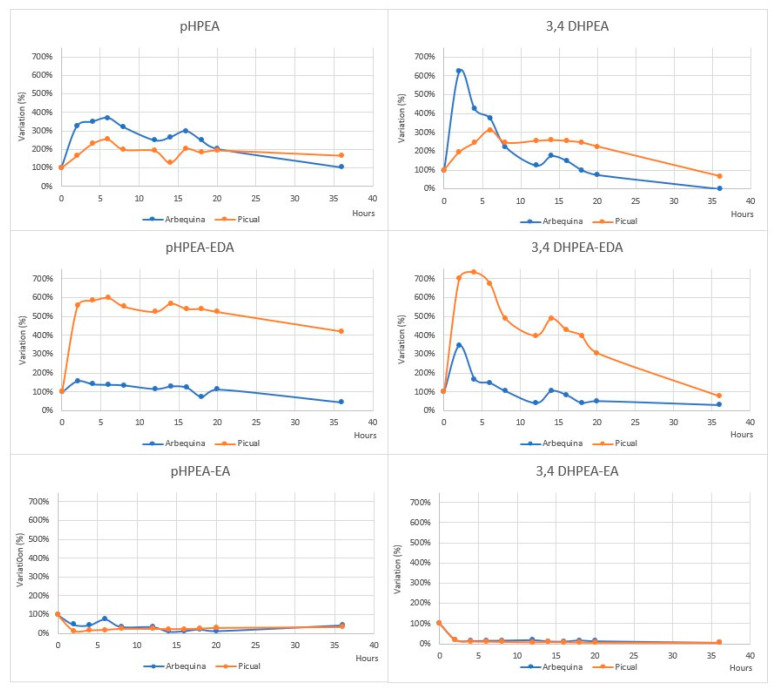
Effects of heating on EVOO phenolic compounds in absence of foodstuff. HPLC relative quantification (with respect of raw amounts) of Ty (**7** in Figure 1), Hy (**8**), 3,4-DHPEA-EA (**18**), pHPEA-EA (**19**), 3,4-DHPEA-EDA (**20**), *p-*HPEA-EDA (**21**) in two oils, with different initial amounts of TPC (82 and 406 mg/kg, respectively, for Arbequina and Picual), subjected to heating at 180 °C for 2–36 h in absence of vegetables. Mean changes were extrapolated from Allouche et al. [61].

Accordingly, Allouche et al. demonstrated that TPC decrease in EVOOs from the same two cultivars (Arbequina with 82 mg/kg and Picual with 406 mg/kg TPC) was higher in proportion in the PC richer EVOO [61]. For example, at both extremes of the analysis (2 and 36 h at 180 °C), residual PC contents were 92% vs. 80% and 53% vs. 36% for Arbequina vs. Picual (data at 2 h was extrapolated from figures of the publication). This is in agreement with the idea that PC are the most effective antioxidants of OO [62] and thus play a role in protecting other components from degradation, i.e., tocopherol, as already suggested 20 years ago [63]. One more interesting point that can be noticed in the data produced by Allouche et al. is the different fate of the EA secoiridoids compared to the EDA ones, especially at 2 h of heating (see Figure 3, partially extrapolated from author publication). Unfortunately, no shorter (cooking-compatible) times were investigated. 

Noteworthy, the data support the fact that the determination of the TPC can be insufficient and misleading for the nutritional evaluation of oils. In fact, while, similarly with Ty and Hy, the EDA secoiridoids (their derivatives linked to the dialdehydic forms of elenolic acid) hugely increased their concentrations, the EA ones (those linked to the mono-aldehydic forms of elenolic acid) were reduced by 60–80%. Both the increase in the EDA secoiridoids and the decrease in EA ones were actually already reported by Brenes [60], who hypothesized the coelution of oxidized forms for the increases; however, such a demonstration is still missing and the authors did not actually consider that the increase in aglycons concentration could simply be a consequence of the breaking of phenol-sugar glycosidic rising phenol concentration [64]. Finally, the differences between Ty and Hy (and their derivates species) showed similar lowering trends, but the lowering of the latter derivatives (the *o-*diphenolic aglycons) at prolonged heating was more marked, which is consistent with their higher antioxidant capacities [65]. 

With respect to other OO nutritionally relevant molecules, pinoresinol and 1-acetoxypinoresinol showed decays with different extents depending on EVOO content, with faster degradation in the phenol-richer EVOO [61]. Conversely, both luteolin and apigenin had opposite behaviour, with faster and higher degradation in the poorer EVOO, even if apigenin showed higher stability [61], accordingly with the lower number of phenolic hydroxyls [66] (Figure 1).

Later, Esposto and co-workers used a refined OO, void of hydrophilic PC, spiked with precise amounts of an already purified and characterized phenolic extract [67], to evaluate the overall and individual fates of PC after 30 min, 1, 2, 4, 6, 8, 10 and 12 h of heating at 180 °C [68]. They found that, even if oils containing higher amounts of PC showed higher resistance to the oxidation of oleuropein derivates and also, notably, the formation of negative volatile compounds and the loss of α-tocopherol, heating induced a huge degradation in PC, proportional with the initial amount [68]. In particular, they found that heating low-phenol EVOO induced the loss of more than 90% of original PC (expressed as the sum of 3,4-DHPEA and 3,4-DHPEA-EDA) already after 30 min, while a similar extent of loss was obtained only after prolonged heating or richer oils, 4 and 8 h, respectively, for oils with 200 and 400 mg/kg of PC [68], which is actually the opposite to what was observed by heating in the presence of water (see Section 4.2.3) or vegetables (see Section 4.3). Notably, they reported time-dependent formation of the oxidized dialdehydic form of decarboxymethyl elenolic acid (ox-EDA), by hydrolyzation of oxidized 3,4-DHPEA-EDA [68], consistent with the lack of accumulation of oxidized and polymerized products of Hy, as previously reported by the same group [69]. Importantly, based on the authors’ data, the preservation of the phenolic fractions and the accumulation of ox-EDA in an EVOO rich in PC (more than 1200 mg/kg) during heating was delayed, compared to that of a refined OO enriched with a similar TPC, but void in *p-*HPEA-EDA, 3,4-DHPEA-EA and *p-*HPEA-EA and lignans, suggesting the higher protecting properties of such molecules and questioning the reliability of the results obtained using refined oils with only individual spiked PC. The authors hypothesize that higher protection could reside on *p-*HPEA-EDA (oleocanthal), actually absent in their phenolic extract, according to the observation that its concentration in EVOO decreased by only 29% after one hour of heating, lasting up to 12 h of heating, compared to that of the double hydroxyl group substances (3,4-DHPEA, 3,4-DHPEA-EA and 3,4-DHPEA-EDA) that were reduced in a time-dependent way and by 80% after 12 h of heating the EVOO. As long as *p-*HPEA-EA is completely degraded after one hour of heating, contribution to ligstroside derivates is supposed to depend exclusively on *p-*HPEA-EDA. Nonetheless, *p-*HPEA-EA had a starting concentration nearly ten-times lower than other secoiridoids [68]. Unfortunately, no data was reported for 3,4-DHPEA-EA, although the authors hypothesize that it may have converted into 3,4-DHPEA-EDA by the opening and decarboxylation of the elenolic ring, a process that could compensate for 3,4-DHPEA-EDA oxidation [68].

#### 4.2.1. Microwaving 

As reported above (Section 4.2), Goulas et al. concluded that microwaving (50 mL for 5 min at 500 W) was the only heating procedure, compared to boiling, frying and baking, that did not induce the loss of TPC (estimated both by Folin–Ciocateau and by fluorimetric assay) [52]. Deeper analysis showed that a concentration-dependent loss of kinetics also occurs by microwaving, but to lower extents. Cerretani et al. [70] evaluated changes occurring to three phenolic fractions (simple PC, secoiridoids and lignans) in different oils and at different times (1.5, 3, 6, 9, 12 and 15 min) of microwaving (90 mL in duplicate, at 720W). Based on their data, in EVOO, the loss starts immediately, while in non-EVOO containing lower concentrations of PC (13 vs. 93 mg/kg), it starts after 6 min of microwaving. However, at the end of treatment (15 min), PC were still present in EVOO (−83%), while in OO they disappeared completely. With respect to single PC, Ty showed higher resistance, which is consistent with what was reported above using heating. Within secoiridoids, 3,4-DHPEA-EDA showed higher resistance, followed by *p-*HPEA-EA and 3,4-DHPEA-EA. Lignans only showed slight decreases, even at the longest times, accordingly with previous results from Brenes [60], however in this case, shorter processes were tested, lower power was used (15 g of oil heated at 0.5 kW for 5 or 10 min) and minor changes were reported for Hy derivatives (20–30% loss) and increases for Ty ones [60]. 

On the other hand, Kishimoto et al. recently reported that microwaving (500 W) induced higher oxidative degradation of EVOO compared to normal heating (200 °C) [55]. As mentioned above, the method used by Kishimoto and co-workers for TPC quantification is poorly validated. Nonetheless, looking deeper at Kishimoto’s data, which used only one oil and valuably examined temperature kinetics, one can see that after 5 min, the loss of PC was double in microwaving (around 29%) than in heating (around 14%), even if microwaved samples reached a lower temperature (140 °C vs. 200 °C). Nonetheless, different starting amounts were used (respectively, 50 and 200 g for microwaving and heating). Thus, weight, time and microwave power are crucial aspects that can deeply affect the final temperature and possibly the fate of PC. In fact, based on data from Cerretani et al. [70], microwaved samples can attain temperatures (315 °C) at the end of the treatments (15 min) higher than those normally obtained with other methods. 

#### 4.2.2. Pan-Frying 

Very recently, Lozano-Castellón and co-workers studied the effects on PC by mimicking the home kitchen process of pan-frying [71]. For such a purpose, they heated a small amount of oil (20 g at 120 or 170 °C for 15 to 60 min) in a pan without controlling oxygen or light, and by means of MS-coupled UPLC they analysed changes occurring in EVOO PC [71]. Unfortunately, they did not repeat the same conditions using a deep-fryer or other oil-cooking techniques, thus their results are not comparable with other methods. In any case, they found that TPC decreased by 40% and 75%, respectively, at 120 °C and 170 °C, and that individual PC had different degradation rates [71]. With respect to longer cooking times, time did not affect TPC loss, which is consistent with previous results showing a similar decrease for 1 h or for 5 h [52]. Notably, they reported that the nutritionally relevant molecule *p-*HPEA-EDA (oleocanthal) was the more resistant compound, similarly with the higher resistance of Ty derivatives compared to Hy ones following heating, as reported by Silva et al. [72] and mentioned above (Section 4.2).

#### 4.2.3. Boiling

Brenes et al. [60] were the first to investigate oil heating in the presence of water (60 g of oil in 2.5 L of water in a pressure cooker for at 109 °C 30 min). They reported “complete hydrolysis” of secoiridoids and “moving” of Ty and Hy to the water phase. However, as data for the oily phase were not reported, leakage could have been overestimated. Accordingly, less important losses for oil heated in presence of water were reported later [72]. Specifically, heating oil in boiling water (60 g of oil in 400 mL of water for 15 or 60 min) was shown to induce higher losses of PC, in proportion to EVOO (27 and 53% loss, respectively, at 15 and 60 min) than from non-EVOO oil (7 and 14%) [72]. Higher resistance of non-EVOO oil was associated with lower losses of secoiridoids, which were almost null except for a 16% decrease for 3,4-DHPEA-EA, even after 60 min of boiling [72], according to other indications of lower stability [61]. Interestingly, based on authors data, even if PC loss was quantitatively higher for EVOO, PC in EVOO showed a higher time-resistance of leaking to the water phase [72]. In any case, between 60 to 80% of secoiridoids leaked to the water phase, increasing from 3,4-DHPEA-EDA > 3,4-DHPEA-EA > *p-*HPEA-EDA > *p-*HPEA-EA. Higher losses observed by Brenes could rely on pressure treatment, in which higher temperature was reached (109 °C). 

#### 4.2.4. Air Baking

Goulas and co-workers [52] reported that hot-air oven baked oil (50 mL at 180 °C for 45 or 90 min) reduced TPC (calculated both by Foline–Ciocateau and by fluorimetric assay) significantly less than heated oil (50 mL at 180 °C for 1 or 5 h). Lower losses by baked (−11%) vs. heated oil (−75%) were not discussed by authors and are actually difficult to explain. However, as poor details were reported on the temperature of samples during processing, the role of the temperature kinetics cannot be excluded. 

Apart from PC with stronger heat resistance (*p-*HPEA-EDA and *p-*HPEA-EA) or simpler PC (Hy and Ty) that remained unchanged, oil roasting (heating in air oven at 180 °C for 1 h, thus actually air baking) was reported more recently to induce a 16% loss of TPC and to significantly alter the phenolic composition [73]. Notably, the addition of vegetables (carrots, potato or/and onion in the 1:1 proportion) was shown to increase PC loss from oil by almost 80% of the initial molecules [73], possibly due to a migration to vegetable that was, unfortunately, not checked by authors (the role of vegetables is discussed below, Section 4.3). Nonetheless, significant alterations, even in the simpler PC Hy and Ty during roasting, in the absence of vegetables, were also reported by other authors [60]. 

### 4.3. Effects of Oil Heating with Vegetables

#### 4.3.1. The Fate of Phenolic Compounds of EVOO during Cooking

As mentioned, vegetables can deeply modify OO PC during heating, and this process is highly influenced by the type of cooking process. One of the main factors affecting cooking performance in terms of protection of PC of oil, but also those of the food matrices, resides on whether the process is carried out in polar or apolar media. In fact, while hydrothermal processes deeply affect hydrosoluble antioxidants, such as PC (see Section 4.2.3), the use of apolar deep or surface frying in theory prevents losses in phenol concentrations of the vegetable matrix.

One of the first works to analyse the fate of PC in the presence of vegetables reported higher susceptibility of dihydroxyphenol components (3,4-DHPEA, 3,4-DHPEA-EDA and 3,4-DHPEA-EA), compared to Ty (*p-*HPEA) and its derivatives (*p-*HPEA-EDA and *p-*HPEA-EA) [74]. The authors deep-fried fresh sliced potato slices (200 g) at 180 °C for 10 min repeatedly with the same EVOO (2 L), twice a day for 6 days, allowing the oil to cool to less than 50 °C between operations, for an overall frying time of 2 h. The authors observed that 3,4-DHPEA and its secoiridoid derivatives rapidly diminished with the number of frying operations (log relationship), with components decreasing by 40–50% of their original concentration already after the first process and by more of 90% after six processes. Even more rapid loss was reported for lignans pinoresinol and 1-acetoxypinoresinol that fell drastically already in the first frying operation. On the other hand, Ty and its secoiridoid derivates showed slower reduction, with a linear relationship with the number of fryings). Higher susceptibility of dihydroxyphenols was attributed to the known higher antioxidant activity of Hy [65,75,76] and, we believe, erroneously (as stated in Section 4.2), with higher degradation in linoleic-richer oil compared to oleic-richer one [60]. 

Later, Casal and co-workers reported that domestic deep frying of fresh potatoes at 170 °C for 3 h induced a loss of almost 50% of TPC of EVOO oil, measured by the Folin–Ciocalteu method [46]. This is actually not surprising, if we consider that heating can result in the loss of original PC in EVOO already after 30 min at 180 °C, as reported above (Section 4.2), rather it seems that the vegetable protects oil PC. However, as the goal of the authors was to determine the degradation effects of long time frying up to 27 h (i.e., till the amount of total polar compounds reached 25%, in accordance to a Portuguese law), shorter times, more typical in home cooking, were unfortunately not tested [46]. Another work studied the relationships between olive ripening and long lasting EVOO stability during up to 40 cycles, 8 min each, of potato-frying [77]. Thanks to the processing of olives from the same cultivar and olive season, although from increasing ripening indexes, the authors tested three EVOOs with decreasing PC content (394, 289, and 78 ppm) and found that greater PC were associated with lower polar compound content and a longer Rancimat induction period [77]. Unfortunately, the negative correlation was reported in an incomplete fashion for the three oils and without detailing its timing throughout the 40 cycles and, above all, was very similar to that for tocopherols.

As mentioned above (Section 4.2.3), Silva et al. [72] reported that boiling in water induced PC loss from non-EVOO oil and, to a greater extent, from EVOO oil. Moreover, they qualified the loss of PC as “dramatic” if heating was performed in the presence of vegetables, i.e., potato, carrot, or onion, in both types of OO, even after only 15 min and for all PC tested, except in the presence of onion, especially from non-EVOO oil [72]. However, based on the authors’ data, as long as the longer incubation time (60 vs. 15 min) results in higher recovered amounts of Hy, one cannot exclude the contribution of the vegetable itself to the molecule, that apparently was not analysed (authors reported quantification only of few molecules, i.e., quercetin-3,4-diglycoside, quercetin-40-monoglycoside, *p-*hydroxyphenylbenzoic acid and chlorogenic acid). On the other hand, authors suggest that increased losses of boiling with vegetables are due to the presence of elements, such as potassium or calcium and also in metals such as iron and copper, which can reduce PC very quickly and therefore contribute to their degradation, as previously reported, especially for Hy [78]. However, as onions can have important amounts of iron and copper (as reported by authors themselves), the higher yield of Hy, specifically and exclusively in samples boiled with onion, appears contradictory.

**Figure 4:**
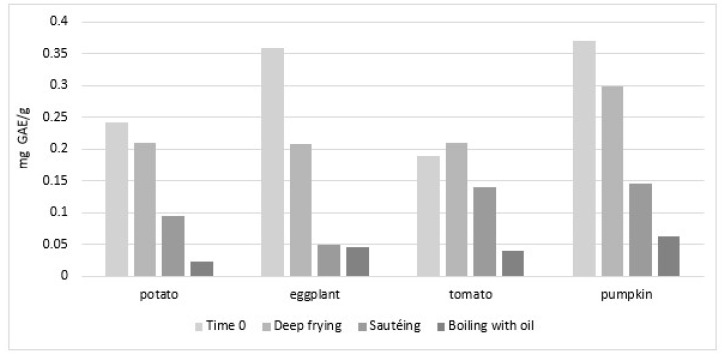
Fate of EVOO TPC following different heating processes in presence of different vegetables. Folin–Ciocalteu absolute quantification of TPC (mg GAE/g of oil) in oil following cooking of vegetables (120 g of potato, pumpkin, tomato or eggplant) for 10 min by deep frying at 180 °C in 600 mL of oil, by sautéing at 80–100 °C with 60 mL or by boiling at 100 °C in 540 mL of water and 60 mL of oil. Mean concentrations were extrapolated from Ramirez-Anaya et al. [79].

The work from Ramírez-Anaya et al. is frequently cited and thus deserves particular attention [79]. The authors studied the fate of individual PC (HPLC) and of TPC (Folin–Ciocalteu) in EVOO following the cooking of vegetables (potato, pumpkin, tomato or eggplant) for 10 min by deep frying at 180 °C (120 g of vegetable in 600 mL of oil) or by sautéing at 80–100 °C (120 g in 60 mL) [79]. They found that both cooking procedures reduced TPC in oil to a greater extent for sautéing, independently on the foodstuff cooked, with the only exception being deep frying in presence of tomato (see Figure 4, built using mean concentrations from [79]). However, as evidenced by differences in TPC at Time 0, the authors probably used different oils; this could explain some of the reported differences. Interestingly, the authors also investigated the fate of TPC in EVOO following boiling in water/oil (W/O, 10 min of heating of 120 g of vegetable at 100 °C in presence of, respectively, 540 mL and 60 mL of water and oil), finding that water increased TPC losses for all vegetable experiments (excluded eggplant, for which losses did not overcome those of sautéing). A limitation of the work is that authors did not perform tests without vegetables in order to assess their contribution to losses of the intrinsic oxidation of PC by heating, or of role of the hydrophilic medium in the migration of EVOO PC.

**Figure 5:**
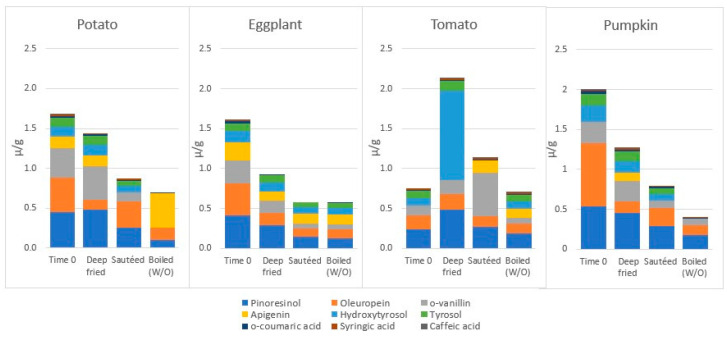
Effects of different heating processes on EVOO phenolic compounds of in presence of different vegetables. HPLC absolute quantification of pinoresinol (**22** in Figure 1), oleuropein (**17**), *o-*vanillin (**6**), apigenin (**14**), Ty **7**), Hy (**8**) and *o-*coumaric (**10**), syringic (**5**) and caffeic (**12**) acids (µg/g of oil) in oil following cooking of vegetables (120 g of potato, pumpkin, tomato or eggplant) for 10 min by deep frying at 180 °C in 600 mL of oil, by sautéing at 80–100 °C with 60 mL or by boiling at 100 °C in 540 mL of water and 60 mL of oil. Mean concentrations were extrapolated from Ramirez-Anaya et al. [79].

With respect to the effect of different techniques on individual PC, authors reported that major losses in PC were induced when vegetables were sautéed or boiled in W/O, down to complete elimination for Hy and Ty by sautéing of tomato and boiling in W/O of potato or pumpkin [79]. With respect to the effect of specific vegetables on EVOO PC, authors reported a net increase in the amount of pinoresinol and Hy in EVOO used to deep-fry tomato, which is different to that reported above for tomato by Gómez-Alonso [74]. However, looking deeper at the authors’ data, the idea that the authors probably used different oils for all vegetable tested appears even more evident, especially for tomato (see Figure 5, that was built with µg/g mean concentrations from data from the publication, considering only molecules detected in all “time 0” oils (i.e., pinoresinol, oleuropein, *o-*vanillin, apigenin, Ty, Hy and *o-*coumaric, syringic and caffeic acids), and this renders the comparison of the effects of vegetables complicated, if not impossible. 

**Figure 6:**
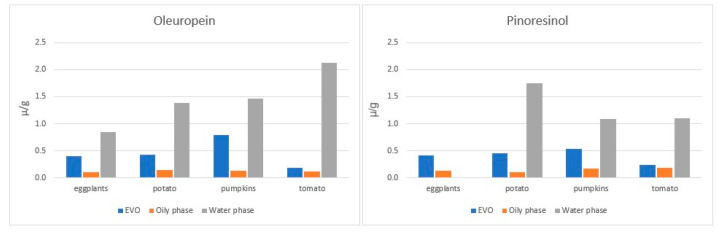
Oleuropein and pinoresinol concentration changes following water/oil cooking of different vegetables. HPLC absolute quantification of oleuropein and pinoresinol (respectively, **17** and **22** in Figure 1) (µg/g) in oily and water phases following cooking of vegetables (120 g of potato, pumpkin, tomato or eggplant) by boiling at 100 °C in 540 mL of water and 60 mL of oil for 10 min. Mean concentrations were extrapolated from Ramirez-Anaya et al. [79].

Nonetheless, looking at the authors’ data on boiling W/O experiments, it appears that the type of vegetable used can significantly affect (based on the statistical analysis provided) the concentration of exclusive EVOO PC in the cooking water (i.e., oleuropein and pinoresinol, thus excluding those that can arise following the hydrolysis of other EVOO molecules, such as Hy and Ty) (Figure 6). However, as authors apparently used different oils, containing possibly different amphiphilic compounds, one cannot exclude that differences in PC migration between the two phases could be due to their interactions in water-oil interfaces [80], rather than on the vegetable cooked. Accordingly, retention of oleuropein following potato frying depends on oil characteristics, as demonstrated by the use of different oils enriched with the same amounts of TPC, as reported in Section 4.1. With respect to lignans, the frying of potatoes induced a strong loss of these molecules, which were instead reported stable in EVOO heated without food (Section 4.2).

The use of spiked PC comes in handy again. As mentioned in Section 4.1, Chiou et al. used an olive leaf extract to enrich olive, sunflower and soybean oils to study the fate of spiked oleuropein during repeated potato frying [49]. The results unequivocally demonstrated that oleuropein is able to migrate to vegetable tissues. Specifically, the authors found that oleuropein content in the oil absorbed by potatoes was found more than ten-times enriched compared to that present in the frying oil, as if the water-rich food tissue protected the migrating relatively polar PC [49] and, with respect of the type of oils, the increase was seventeen, sixteen, and thirteen times higher in average for sunflower, olive and soybean oils, respectively [49]. 

**Figure 7:**
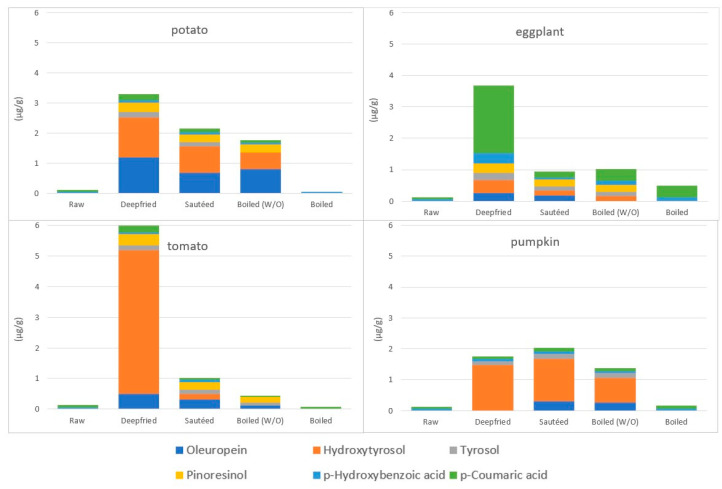
Incorporation of EVOO individual phenols in vegetables following cooking. Folin–Ciocalteu absolute quantification of oleuropein (**17** in Figure 1), Ty (**7**), Hy (**8**), pinoresinol (**22**), *p-*hydroxybenzoic (**1**) and *p-*coumaric (**11**) acids (µg/g) in vegetables (120 gr of potato, tomato, eggplant or pumpkin) before and after cooking for 10 min in 600 g of EVOO at 180 °C by deep-frying or with 60 g of EVOO at 80–100 °C by sautéing, or in 60 g oil plus 540 gr of water by water/oil (W/O) mixture boiling at 100 °C or in 600 g of water at 100 °C. Mean concentrations were extrapolated from Ramirez-Anaya et al. [79].

De Alvarenga and co-workers recently studied the migration of PC from EVOO and vegetables during the preparation of the highly widespread Mediterranean cooking practice of tomato sauce consisting in two steps, 1 min frying of onion (400 g) and garlic (40 g) in 100 g of EVOO sofrito, followed by the addition of 460 g of tomato and further heating for 30 min at 100 °C [81]. Specifically, authors found that, among the eight phenolic molecules already present and detectable in raw EVOO by means of UPLC-MS (apigenin, elenolic acid, ferulic acid, ligstroside, luteolin, *p-*coumaric, oleuropein and pinoresinol), all were considerably reduced in the oily phase of the sofrito, apart from one that remained unchanged (pinoresinol) and one, i.e., ferulic acid, that was considerably enriched (by more than 15 times), possibly coming from garlic and tomato [81]. The migration of PC from EVOO to vegetables was indeed demonstrated by the quantification of individual EVOO PC (oleuropein, Ty, Hy, pinoresinol, *p-*coumaric and *p-*hydroxybenzoic acids) in vegetables following cooking with different techniques, as shown in Figure 7, extrapolated from Ramírez-Anaya’s data [79]. Notably, the authors were able to demonstrate the enrichment of the vegetable’s profile with specific EVOO PC, also by the W/O boiling technique.

**Figure 8:**
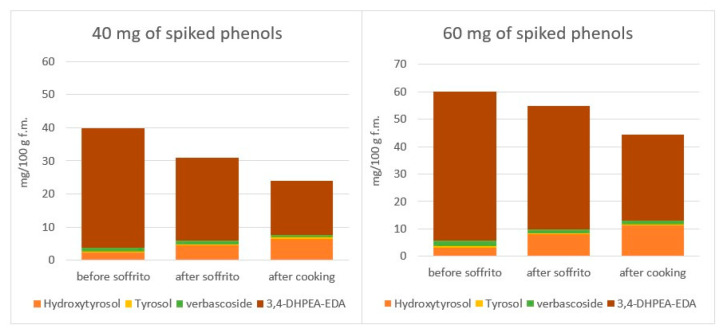
Fate of spiked phenols during tomato sauce preparation with refined oil. Figure 8 HPLC absolute quantification of Hy (**8** in Figure 1), Ty (**7**), 3,4-DHPEA-EDA (**20**) and verbascoside (**23**) (mg/100 g f.w.) following sofrito (celery, onion and carrot) and tomato passata cooking with a refined OO spiked with an extract containing indicated phenols at two different concentrations. Mean concentrations were extrapolated from Taticchi et al. [82].

**Figure 9:**
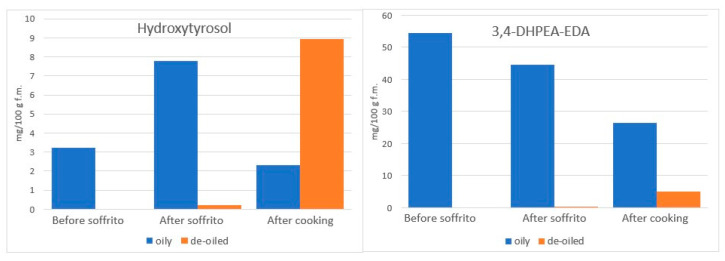
Migration of spiked Hy and 3,4-DHPEA-EDA during tomato sauce preparation with refined oil. HPLC absolute quantification of spiked Hy and 3,4-DHPEA-EDA (respectively, **8** and **20** in Figure 1) following sofrito (celery, onion, and carrot) and tomato passata cooking with a refined OO spiked with an extract containing indicated phenols at two different concentrations. Mean concentrations were extrapolated from Taticchi et al. [82].

The fate of PC during sauce preparation was also previously studied by the group of Servili [82], whose work we have already mentioned [68] in the paragraph dealing with heating without vegetables (Section 4.2). Similarly, with their previous work, the authors spiked an extract of PC (containing Hy, Ty, 3,4-DHPEA-EDA and verbascoside) in 100 g of refined oil to be used for a sofrito preparation, obtained by heating at 100 °C chopped fresh celery, onion and carrot (20 g each) for 10 min, followed by the addition of tomato passata (800 g) and further boiling at 100 °C for 20 min. As expected for the presence in the extract of 3,4-DHPEA-EDA, the entire process induced an increase in Hy (but not Ty) concentration consistent with the loss of its derivative, as shown in Figure 8, which was built summing the absolute mean amounts of the 4 phenols from concentrations reported in the publication and obtained from two different amounts of spiked PC. Notably, based on authors data on the concentrations of individual PC in the oily and non-oily phases, Hy increase took place during sautéing but apparently migrated from the oily to the non-oily phase only after the addition of tomato, while the decrease in 3,4-DHPEA-EDA was not associated with the equivalent migration (Figure 9). Moreover, by spiking two concentrations of PC, the authors demonstrated that the loss of PC is not linear with the initial concentration (spiking with 60 mg induced lower-in-proportion loss compared to spiking with 40 mg), both after sautéing (8.6 vs. 22.2% loss, respectively) and after tomato cooking (26.2 vs. 39.9%) [82], which is opposite to what was observed by heating without vegetables (Section 4.2). Notably, the protective concentration-dependent effect of EVOO PC was also observed on specific PC in vegetable ingredients (quercetin-3-*O*-rutinoside, apigenin and luteolin), and on their carotenoids and α- tocopherol [82]. In particular, based on the authors’ data, it appears that the more EVOO contains TPC, the less they will be lost by sautéing and tomato cooking.

#### 4.3.2. The Incorporation of Phenolic Compounds of Vegetables in EVOO during Cooking

In the above-mentioned work on tomato sauce by de Alvarenga et al., even if the net increase in TPC in EVOO was not quantified, the authors demonstrate that cooking can enrich EVOO in individual vegetable-specific PC [81]. In fact, the oily fraction of the sofrito was enriched by molecules not present in raw EVOO, namely caffeic acid, caffeic acid hexoside, chlorogenic acid, naringenin, 3*,*4-dihydroxybenzoic acid, and quercetin, possibly thanks to the cooking-induced hydrolysis of progenitor phenolic glycosides yielding free hydroxylphenols groups with increased solubility in oil [81]. Notably, at the end of the process, the oily fraction of sofrito contained more PC than the water one, especially naringenin, whose concentration was similar to that present in raw tomato [81]. Authors ascribe the enrichment of sofrito in PC mainly to the step of frying onions, possibly yielding naringenin and ferulic at amounts consistent with increased absorption from tomato sauce cooked with OO, compared to tomato sauce cooked without OO (see below the work from Lamuela-Raventos’ group, in the bioavailability Section 4.4.2). Unfortunately, the authors did not perform a control sofrito, skipping the onion frying step without omitting EVOO, which would have corroborated that enrichment was to be ascribed to EVOO and not to cooking.

**Figure 10:**
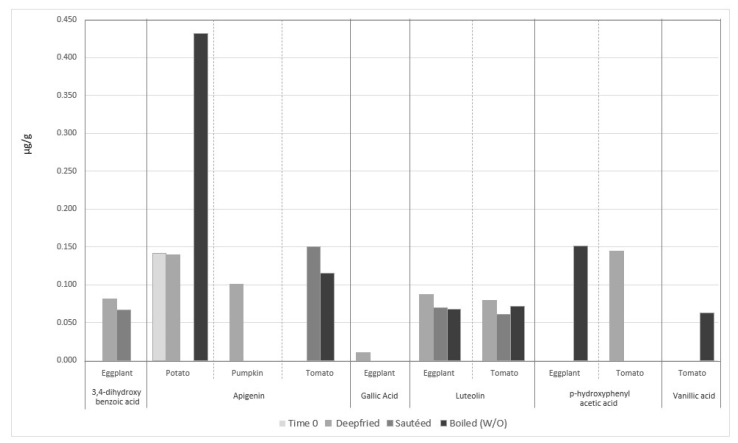
Incorporation of individual vegetable phenols in EVOO following deep frying, sautéing and boiling in water. HPLC absolute quantification of apigenin (**14** in Figure 1), luteolin (**15**) and 3,4-dihydroxybenzoic (**2**), gallic (**3**), *p-*hydroxyphenyl acetic (**9**), and vanillic (**4**) acids (µg/g of oil) in oil before and after cooking of vegetables (120 gr of potato, pumpkin, tomato or eggplant) for 10 min by deep frying at 180 °C in 600 mL of oil, by sautéing at 80–100 °C with 60 mL or by boiling at 100 °C in 540 mL of water and 60 mL of oil. Mean concentrations were extrapolated from Ramirez-Anaya et al. [79].

Ramírez-Anaya and co-workers, already cited for their results on the fate of PC in EVOO following heating (Section 4.3.1), followed also the incorporation of individual PC of vegetables in EVOO, following deep frying, sautéing and boiling in water [79]. Although they did not quantify PC in vegetables, they reported that out of 14 vegetable molecules, five appeared and one increased their concentration in EVOO following cooking, compared to unused oils, i.e., 3,4-dihydroxybenzoic, gallic, *p-*hydroxyphenyl acetic and vanillic acids and luteolin and apigenin (see Figure 10 showing absolute mean concentrations from authors publication). Notably, the authors show that when using a heat matrix, a mixture of water and oil (boiled (W/O), PC migrated more and mainly towards the water phase.

Such studies are relevant as they can help to define the rules for the predictability of PC migration during different cooking procedures. However, from a nutritional point of view, as no one would heat the residual frying oil (unless in the obvious case of sautéing), the study of the migration of PC to the opposite direction, i.e., from EVOO to vegetables, is trivially more relevant.

#### 4.3.3. The Incorporation of EVOO Phenolic Compounds in Vegetables during Cooking 

**Figure 11:**
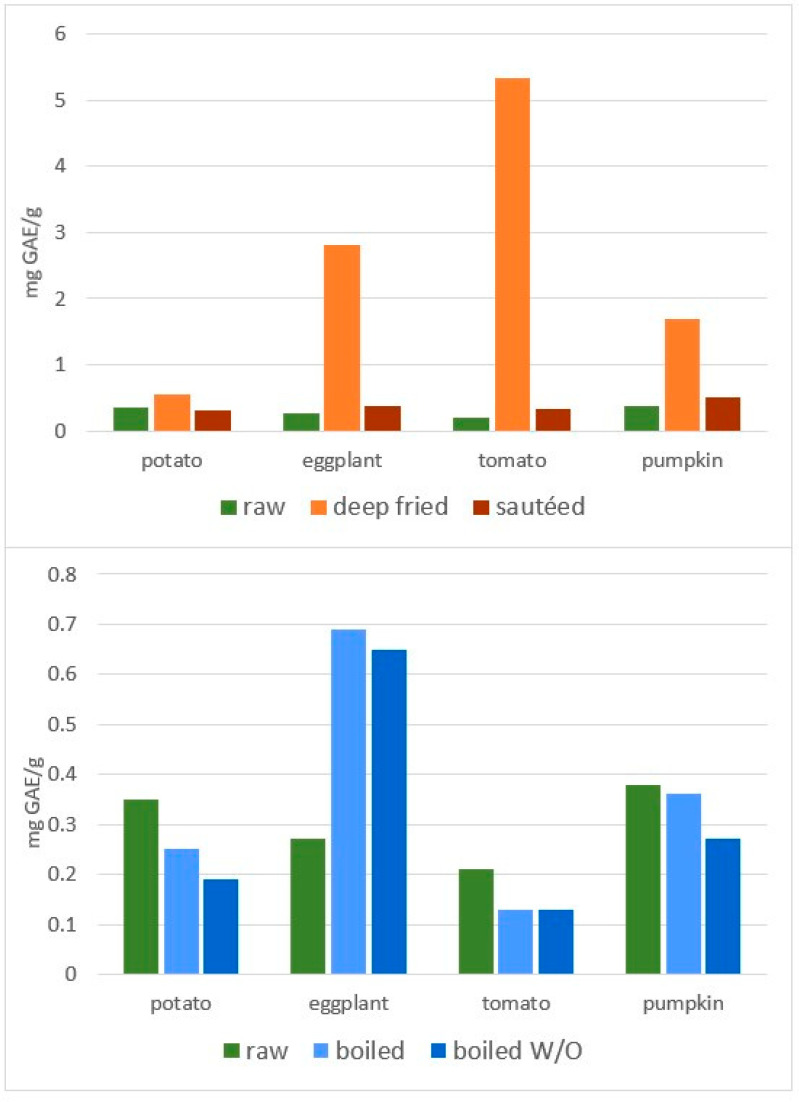
Incorporation of EVOO TPC in vegetables following cooking. Figure 11 Folin–Ciocalteu absolute quantification of TPC (mg GAE/g) in vegetables (120 g of potato, tomato, eggplant or pumpkin) before and after cooking for 10 min in 600 g of EVOO at 180 °C by deep-frying or with 60 g of EVOO at 80–100 °C by sautéing, or in 60 g oil plus 540 g of water by water/oil (W/O) mixture boiling at 100 °C or in 600 g of water at 100 °C. Mean concentrations were extrapolated from Ramirez-Anaya et al. [79].

Reports investigating the migration of EVOO PC in vegetables following EVOO-cooking yielded contradictory results. In fact, in the work from Kalogeropoulos et al., a net transfer of PC from EVOO to vegetables (potato, green pepper, zucchini and eggplant) was reported by sautéing, despite the fact that both EVOO and vegetables had lost between 30 to 75% of their own TPC [50]. Specifically, based on the authors’ data, chlorogenic acid increased its concentration by six times in green pepper (from 0.1 to 0.6 mg/100 g), accounting largely, according to the authors, for the “retention” of EVOO PC in the vegetable. However, as the molecule had a significantly lower concentration in fresh EVOO (0.02 mg/100 g), it looks unlikely that chlorogenic acid found in fried green pepper originated from EVOO. An increment in the TPC content of vegetables following pan-frying (and, to some extent, following sautéing) with EVOO was reported later, also by Ramírez-Anaya and co-workers [79]. Specifically, authors quantified PC in 120 g of diced (1 cm^3^) potato, tomato, eggplant or pumpkin, cooked for 10 min in 600 g of EVOO at 180 °C by deep-frying, or in 60 g of EVOO at 80–100 °C by sautéing, or in 60 g oil plus 540 g of water by W/O mixture boiling at 100 °C (Figure 11). According to the authors’ data, deep frying significantly increased TPC concentration in all four vegetables. As shown in Figure 11 extrapolated from the authors’ data, the highest increase in TPC concentration following deep frying was for tomatoes (25 times more), followed by eggplant (10 times), pumpkin (almost five times) and potatoes (57% more). As no indication was reported on the amount and types of PC present in oil used, one cannot exclude a migration effect from oil. However, the quantification of TPC in W/O-boiled vegetables indicates the poor contribution of EVOO to vegetable enrichment of TPC (Figure 11). In fact, boiling vegetables in the W/O mixture led to a slight loss of PC from all vegetables except eggplant, whose increase was, however, oil-independent, as it was also found in water-boiled vegetables (120 g of each vegetable in 600 g of water at 100 °C). A possible explanation comes from erroneous quantification of TPC by the Folin–Ciocalteu method, or more specifically to the observation of deep changes in fat (increased) and moisture (reduced), correlating with TPC changes [83]. These observations indicate that the correct evaluation of the chemical transformations, possibly occurring in vegetables following sautéing or frying, requires parallel evaluation, not only of the effects of heating on oil itself (oil heating alone, without vegetables), but also of those on vegetables using similar conditions but without oil, for example in an oven. In fact, such controls could have indicated a change in the accessibility of PC at the end of the heating procedure. Nonetheless, as reported above, the authors, in spite of this, were able to demonstrate some migration of PC from EVOO to vegetables (see Section 4.3.1 and Figure 7) [83].

#### 4.3.4. The Fate of Phenolic Compounds of Vegetables during EVOO-Cooking 

Recent reports indicate that oil cooking techniques induce higher losses in the PC of vegetables compared to other oil-free cooking techniques. For example, while microwaving and boiling induced negligible TPC losses, significant losses were reported in red cabbage for stir-frying [84] and in purple-fleshed potatoes for frying, stir-frying and air-frying [85]. Specifically, Tian et al. showed that the decrease in TPC was the highest for stir-fried potatoes (72.44% reduction). However, the comparison is worth little, as stir-frying was performed with potatoes cut eight-times thinner compared to other frying techniques. In this regard, the authors also tested the new technique of air-frying, using three-times less fat compared to frying (10 mL of soybean oil for 300 g of potatoes instead of 3 L for the same quantity of potatoes), finding a higher reduction of TPC (32.52% reduction) compared to frying (14.08%), possibly because air-frying lasted longer (18 min at 180 °C instead of 2 min at 191 °C for frying). Before concluding that air-frying is a healthier frying technique, based only on the fact that less oil is used, one should also consider that it induces more than doubled the reduction of TPC [85]. Similarly, even if PC were not quantified in the white coconut oil used, Gunathilake et al. reported that frying induced, in six different edible leaves, higher losses of TPC and flavonoids compared to boiling and steaming, and the extent of the losses depended on the vegetable species [86]. On the other hand, Mashiane and co-workers reported that within Cucurbitaceae stir-frying determined lower losses than microwaving [87]. However, authors stir-fried pumpkin leaves using an EVOO (10% volume compared to the weight of leaves), the PC of which were not quantified.

With respect to a single PC, in their already repeatedly mentioned article, Silva et al. reported that frying for 15 min in EVOO conserved most of onion and carrot quercetin and *p-*hydroxyphenylbenzoic, respectively, while potatoes kept only small amounts of their chlorogenic acid [72], accordingly with previous reports, showing that frying did not affect quercetin concentration in onion, while boiling induced 30% loss of glycosides [88]. Similarly, Jung et al. [89] performed an analysis of the fate in the roots of sweet potato cooked with six different home-processing techniques (boiling, deep frying, microwaving, oven baking, sautéing, and steaming) of six chlorogenic acids, demonstrating that, in general, deep frying showed the greatest reductions overall. Notably, authors from the same group subsequently used the fate of these PC to set up a functional mathematical index for predicting the effects of food processing [90]. Some works indicate apparent opposite effects. Martini and co-workers reported a significant increase in eggplant total hydroxycinnamic acids concentration following all tested cooking techniques (baking, boiling, frying and grilling), and a higher increase for sunflower vs. OO [91]. Similarly, for onion, green pepper or cardoon, identical frying conditions almost doubled the concentrations of chlorogenic acids and flavonoids in cooked vegetables, with respect to raw ingredients, using sunflower but not olive oil. However, even if higher temperatures were used (150 instead of 115 °C), increases in chlorogenic acids were even higher for griddling without oil [92]. Managa et al. reported that stir frying (at 125 to 140 °C) Chinese cabbage leaves with EVOO (10 mL) in nightshade (120 g) increased their concentration of kaempferol and quercetin derivatives and caffeoylmalic and chlorogenic acids, especially the latter [93]. However, similar to Martini’s results, concentrations also increased (doubled with respect to raw) with other cooking techniques tested, with overall similar yields for microwaving, boiling and steaming, but lower than stir frying (quadrupled), supporting the general role of heating in also increasing individual PC accessibility in some specific vegetables (accessibility should not be confused with bioaccessibility, see Section 4.4.1). Accordingly, changes in vegetable matrices or enzymes were associated with a steaming or boiling-dependent increase in TPC content in artichoke leaves [94]. Similarly, a comparison of the effects of cooking treatments (boiling, steaming and microwave-cooking) of several Mediterranean wild edible species without employing oil, reported that any kind of heating increased the accessibility/availability of chlorogenic acid and rutin, and this could possibly also contribute to the net increase in TPC associated with any cooking procedure [95]. 

### 4.4. Changes in Bioaccessibility and Bioavailability of Vegetable PC Following Cooking with Oil

A terminological explanation is needed. Normally, bioaccessibility is related to digestion and absorption efficiencies of a nutrient, or any bioactive compound orally administered. It is normally expressed as the ratio between the amount of the constituent released and absorbed and its total amount ingested, regardless of whether the body is then able to use it in its metabolic processes. Basically, bioaccessibility depends on events driven by (1) digestion (that makes the bioactive molecules potentially bioaccessible matter); (2) absorption through epithelial tissue; and (3) pre-systemic metabolism. The term bioavailability is wider as it also includes the nutritional efficiency of the bioactive compound, i.e., the capability of the body to really “use” and take advantage of a particular component. Thus, bioavailability depends firstly on the systemic distribution of the compound and secondly on its potential interaction with target tissues. It is expressed as the ratio between the amount of the constituent actually utilized in metabolic functions (or stored to be subsequently used in metabolic functions) and the total amount introduced with the diet.

Several studies have been published to describe the bioaccessibility and bioavailability of PC in different food matrices. The review published by Shahidi and Peng cited 356 references and concluded that the bioaccessibility and bioavailability of different PC strictly depends on the effective dose taken, the general physical condition of the individual, and on the activity and efficiency of various internal mechanisms (i.e., digestion, absorption, transport, metabolic processes, excretion and microbiota activities) [96]. In fact, no more than 10% of the total intake of dietary PC are directly absorbed and almost all are metabolized in the gut microbiome to more easily absorb metabolites [97]. However, some recent indications support the possibility that an increase in the absorption of PC could be mor easily improved by their incorporation into OO.

Obviously, the thermal treatment of any cooking process may also modify the structure of the bioactive molecules present in the food, which may consequently influence bioaccessibility and bioavailability. In general, it can be stated that the cooking process and/or the heat treatment exerts a positive effect on bioaccessibility and bioavailability due to the softening of the cells [98]. Moreover, several cooking procedures involve the addition of other ingredients that can positively or negatively influence the bioaccessibility and bioavailability of all the components. A typical example is the culinary use of OO, which is known to improve the bioavailability of lipophilic compounds (such as carotenoids) by acting as food excipients and enhancing their extraction [99].

Unfortunately, only few papers among those dealing with the effects of thermal treatment of PC of OO, have measured changes in bioaccessibility and bioavailability. Moreover, most are in vitro studies or were performed on a very small number of subjects. For that reason, very little is known about that matter, and even less is known about the impact of a vegetable foodstuff-OO combination during processing on PC bioaccessibility and bioavailability. 

#### 4.4.1. Bioaccessibility 

Very few and inconclusive studies have been published on the influence of cooking on the bioaccessibility of PC, in part due to inaccurate terminology. In fact, as mentioned in the above paragraph, studies on bioaccessibility should at least be performed though in vitro simulated gastrointestinal digestion, in this case defining bioaccessibility as the ratio between PC contents after and before in vitro digestion. Thus, higher than 100% values of bioaccessibility are not expected and, if reported, probably reflect incorrect extraction or quantification, due to, for example, the formation of water-soluble Maillard reaction products during cooking [100]. This is the case for the already reported work in Section 4.3.4 from Martini and colleagues, which compared the effects of different cooking techniques on the stability of dark purple eggplants PC. In particular, the authors reported higher than 100% values, possibly because they calculated bioaccessibility using PC concentration of a methanolic extract instead of that of the starting tissue. In any case, in relative terms, compared to other cooking techniques, they found the lowest bioaccessibility for frying [91]. Juàniz and colleagues measured the effect of griddling cardoon or frying it in olive or sunflower oil. The author reported that only 2% of PC were bioaccessible in raw cardoon, whereas in cooked samples, up to between 60 and 67% of TPC remained bioaccessible after gastrointestinal digestion, with griddling showing the best performance [101].

#### 4.4.2. Bioavailability 

Excluding studies performed on cereals, the heat treatment for which is carried out without the use of a fat matrix, papers available in the scientific literature dealing with PC bioavailability mainly refers to the preparation of the tomato sauces and sofrito prepared with tomatoes, onion and VOO or EVOO, classic condiments for the preparation of typical dishes of the Mediterranean diet. Worth a mention is the fact that, regardless of the effects of the presence of oil, the results about the increase in bioavailability of tomato PC following cooking were already known as early as 2004, for example for naringenin and chlorogenic acid [102]. Among them are three randomized controlled cross-over studies performed by the work led by Lamuela-Raventós. In the first pilot randomized controlled cross-over study, scholars studied the effect of the addition of an oil matrix during tomato sauce processing, on the accessibility/extractability and bioavailability of 11 PC of tomato [103]. The authors found that cooking increased the accessibility or extractability of 9 out of 11 compounds (in particular naringenin, rutin and ferulic acid); however, that was never improved by the presence of 5% oil, which is somehow unexpected, considering similar previous work showing increased yield when using 10% instead of 5% EVOO [104]. Indeed, oil presence decreased accessibility or extractability for about half of them compared to the sauce cooked without oil. Nevertheless, the presence of OO (EVOO or refined) in the preparation of tomato sauces increased plasma concentrations in naringenin glucuronides, but not in a statistically significant way, possibly due to high individual variability among very few subjects (*n* = 5). Notably, ingestion of the oil-enriched tomato sauces was associated with re-absorption events of PC, possibly induced by a lipid matrix-stimulated enterohepatic circulation [103]. Interestingly, based on the authors’ data, pharmacokinetics differences were also noticed for naringenin between EVOO and refined oils. Unfortunately, the authors did not characterize PC nor fat present in the two oils, as protecting or influencing tomato PC, which could have at least partially explained the observed differences. Three years later, the group investigated the same molecules in another randomized controlled study (carried out on eight subjects), using only a refined oil void of any phenol and characterized for fat content, obtaining different results for oil presence during tomato cooking, i.e., statistically not significant increased accessibility or extractability for some PC (especially naringenin and caffeic acid hexose) but no effects on bioavailability [105]. The increase in naringenin could depend on its ability to be released from the cuticle of tomato where it is trapped. In their last controlled, randomized crossover feeding trial, performed with a significantly higher number of subjects (*n* = 40), the group demonstrated the higher bioavailability of naringenin glucuronide and quercetin, even in presence of statistically not significant increases in PC concentration, following oil addition in tomato cooking [106]. Again, the authors did not characterize the refined oil for PC content nor fat. The use of an EVOO with known amounts of PC or, better, of a refined oil with defined amounts of added PC, could have added more information on PC ability to protect tomato PC and increase their bioavailability. 

Some other experiments studied the vascular protective [107] or insulin sensitivity properties [108] of the tomato-based sofrito in obese rats. However, even if PC were quantified in experimental food administered to animals, the nutrients of the tomato-based sofrito were divided into specific diets such as to allow to discern the effects of oil and/or PC. Unpublished data from the review by Garcimartín and co-workers support different effects of fried oils in rat jejunum, i.e., increased antioxidant enzymes following administration of discontinuously used EVO compared to sunflower [10]. However, to our knowledge, no results have been made available so far. Nevertheless, some data indicate that the beneficial effects of sofrito on rats could be attributed to PC [107,108]. Unfortunately, these molecules were quantified in experimental food administered to animals only in one publication [107].

## 5. Conclusions

The analysis of the literature of TPC fate during oil cooking appears at times conflicting. Some contradictory results can be explained by the poorly pertinent usage of terminology on cooking procedures, different protocols adopted in the different studies aimed at the same objective, different types and quality of oils used in the experiments, and different quality and quantity of PC present in the used oils and in the studied vegetables. For example, despite there being several experimental indications (also in food-less studied) that PC prevent tocopherol degradation during frying, more experiments are needed in order even to conclude to what extent PC, and not tocopherols, are relevant for maintaining fried oil shelf life. Obviously, the type of cooking (mainly its temperature and duration), deeply modifies the status and the composition of the food as a whole, beyond the sole presence of PC, and this concept should be taken into consideration when selecting a procedure to cook our foods. With respect simply to the PC fate, it is clear that the strategy adopted to minimize the loss of PC should account for a low cooking temperature and short cooking time (less than 15 and 6 min, respectively, for standard and microwaving heating time processing, to prevent the loss of the healthy but less stable OO secoiridoids 3,4-DHPEA-EDA and 3,4-DHPEA-EDA). Regarding the frying techniques, results are hardly comparable as a result of the different incubation times, which were typically shorter for frying. Regarding the type of oil, EVOO is basically better than OO, except in frying and apparently in microwaving, as higher TPC content does not confer increased PC stability. With regards to water/oil cooking, the choice should depend on the raw ingredient to be cooked. In general, when the PC content of the raw vegetable is high, cooking in the presence of water will not significantly affect PC concentration. However, the consumption also of the cooking water would benefit their uptake. On the other hand, when content is low, sautéing and frying with EVOO can compensate for the foodstuffs’ weaknesses or enrich them in PC.

Overall, if one wants to accurately compare the effects of a particular cooking procedure in the presence of a certain vegetable (or water or other polar/apolar media), on the biological activity of (to simplify) one PC of EVOO, he should (1) use the same oil (or oils for oil-oil comparisons) and oil/food ratios for all heating treatments; (2) independently subject the oil and the vegetable to similar heating (temperature, time, pressure, evaporation) and processing (dicing, slicing) treatments; (3) run parallel experiments with other cooking techniques using at least one identical physical condition (for example the same temperature for deep- and air frying, or, for microwaving, thoroughly following the heating kinetics of the apparatus); (4) use parallel oil samples with quantitatively precise spiked amounts of the purified PC and a spiked refined oil with no PC at all; (5) preliminarily assess the recoverability of spiked PC in raw conditions; (6) analyse the kinetic of losses/accumulation of both individual PC and their derivatives (for example oleuropein and the four secoiridoids, their oxidized/hydrolysed forms, and Ty and Hy, by HPLC or MS) and TPC (Folin–Ciocalteu or another validated method) in both oil and vegetable heated together and separately (especially for sautéing), starting with short incubation times (minutes) and up to home-compatible usages (up to one hour) or industrial (days with/without cycle replenishments); (7) in order to assess the role of oil tocopherols, tocotrienols, pigments, minerals or fats or other molecules, analyse their content; and (8) do not confuse accessibility with bioaccessibility and bioavailability.

From a nutritional point of view, it is worth mentioning that, thanks to its light frying conditions in EVOO, the sofrito obtained sautéing tomatoes, carrots and onions at low temperature and for a short time length, is actually the base for many Mediterranean dishes and recipes and has been included in a validated questionnaire used to evaluate adherence to the Mediterranean diet [109]. As indicated by the transfer from oil to vegetables of molecules that are exclusively found in EVOO (for example oleuropein derivatives, pinoresinol, Ty and Hy), frying could also be viewed as a potential nutritional solution to increase the uptake of PC. The question remains as to how relevant this enrichment could be from a healthy point of view. In terms of uptake, experiments concerning the controlled enrichment of EVOO indicate that normal amounts of PC in EVOO are still enough concentrated to theoretically provide bioactive molecules at amounts claimed to lower blood LDL oxidation and thus the risk of cardiovascular diseases [110], i.e., at the minimum level of supplementation of 5 mg per day [82]. However, as analyses of bioavailability are very limited, controlled clinical trials are crucial for confirmation. Moreover, experimental data strongly suggest that the amount of PC transferred in food is proportional with the total amount of oil being absorbed by food. Defining a nutritional goal by just increasing the PC intake without considering the composition of the ingested food, as a whole, is nutritionally meaningless. One should consider: (1) the uptake of fat, (2) whether such fried EVOO is healthy, (3) whether there other unwanted compounds, and (4) if this procedure economically advantageous. Accordingly, the guidelines of all western countries concerning healthy nutrition advise against the frequent use of deep frying, in order to limit the ingestion of oxidized fatty acids and/or undesirable compounds (i.e., heterocyclic amines, acrylamide, acrolein, hexanal etc). Thus, the authors that speculated that frying with EVOO could improve the dietary fatty acid profile of ingested food thanks to the presence of PC [10] should consider that the dietary fatty acid profile would also equally improve with the use of less violent and more healthy cooking procedures or, even better, by using raw EVOO in our dishes.
